# OTSUCNV: an adaptive segmentation and OTSU-based anomaly classification method for CNV detection using NGS data

**DOI:** 10.1186/s12864-024-10018-6

**Published:** 2024-01-30

**Authors:** Kun Xie, Xiaojun Ge, Haque A.K. Alvi, Kang Liu, Jianfeng Song, Qiang Yu

**Affiliations:** 1 School of Computer Science and Technology, Xidian University, Xi’an, 710071 China; 2 Hangzhou Institute of Technology, Xidian University, Hangzhou, 311200 China

**Keywords:** Copy number variation, Next-generation sequencing, Adaptive segmentation, OTSU, Anomaly detection

## Abstract

Copy-number variations (CNVs), which refer to deletions and duplications of chromosomal segments, represent a significant source of variation among individuals, contributing to human evolution and being implicated in various diseases ranging from mental illness and developmental disorders to cancer. Despite the development of several methods for detecting copy number variations based on next-generation sequencing (NGS) data, achieving robust detection performance for CNVs with arbitrary coverage and amplitude remains challenging due to the inherent complexity of sequencing samples. In this paper, we propose an alternative method called OTSUCNV for CNV detection on whole genome sequencing (WGS) data. This method utilizes a newly designed adaptive sequence segmentation algorithm and an OTSU-based CNV prediction algorithm, which does not rely on any distribution assumptions or involve complex outlier factor calculations. As a result, the effective detection of CNVs is achieved with lower computational complexity. The experimental results indicate that the proposed method demonstrates outstanding performance, and hence it may be used as an effective tool for CNV detection.

## Background

Copy number variation is a type of structural variation in which a copy or deletion event impacts a large number of base pairs. According to evidence, copy number variations in specific genes may affect the levels of gene expression in one or more cancer types, which may affect how many types of cancers develop and progress [1]. Deletions or amplifications of relatively significant DNA fragments are referred to as copy number variations (from 50 base pairs to several trillion bases) [2]. Because of the intimate relationship between CNV and gene expression, particularly in the tumor [3] cells where the influence of CNV on oncogenes and suppressor genes is particularly significant, as well as the high association of specific copy number variants with intellectual disability, autism [4], and schizophrenia [5], detecting CNV has become an important challenge for researchers and clinical laboratory practice.

More and more CNV detection techniques are being developed as a result of the advancement of next-generation gene sequencing technologies and the expansion of the volume of data produced [6]. These techniques generally fall into one of four categories for data utilization: paired-end mapping (PEM), read depth (RD), split read (SR), and de novo genome assembly (AS). Each of these approaches has unique traits and a range of potential applications. The PEM-based method, which has a superior identification effect for big-length deletion, employs the relation between the spacing of the double-ended read segment and the length of the inserted fragment to assess whether the gene sequence is altered. The SR-based method detects variant breakpoints using non-normal alignment information and offers good detection results for all deletion lengths. The AS-based strategy to reassemble short sequences before variant identification, which theoretically should have the greatest identification results, is seldom used in real investigations because of the enormous amount of good quality data that is needed as well as the expensive cost of assembly. The primary method for identifying genomic copy number variations is the RD-based approach, which relies on the correlation between read coverage depth and actual copy number [7–9]. Theoretically, it is capable of identifying any type of variation, but due to coverage depth’s statistical properties, it needs to be enhanced in terms of its ability to detect copy number variation that is smaller in size and amplitude.

Based on the NGS data and the aforementioned methodologies, several different methods have been created, the majority of which are RD-based. FREEC [10, 11] calls genomic alterations by constructing and normalizing read depth profiles, it can also estimate the purity of tumor cells and can be used for the detection of germline variant events when control samples are provided. CNVnator [12] detects copy number variation events by employing a mean-shift algorithm on read depth profiles under a predefined strategy. ACE [13] fits a model to the read depth data, calculates the tumor purity and cell ploidy with the least amount of error, and then forecasts the absolute copy number. iCopyDAV [14] detects copy number variation events utilizing Total Variation Minimization (TVM) and Circular Binary Segmentation (CBS). CNV-LOF [15] finds CNVs from the standpoint of local data density, which significantly improves the efficiency of local CNV identification. CNV_IFTV [16] creates isolated forests to calculate the anomaly scores of read depth profiles, then applies the total variation model to smooth the scores and forecast CNVs. By combining different sequencing signals, LUMPY [17] suggests a signal mapping framework to predict CNV. It can also find several other forms of gene structural variants. PEcnv [18] fills the gap in the recognition of small CNVs by detecting CNVs of varying sizes using a base coverage corrected model and a dynamic sliding window. IhybCNV [19] improves detection performance by integrating results from different detectors. LDCNV [20] blends global and local and presents a better anomaly score computation algorithm based on KNN that more accurately captures the degree of abnormality. Restricted by the intrinsic complexity of NGS data, how to efficiently retrieve valuable information from the heavy data and how to set thresholds with more confidence still has to be researched further to further evaluate the data features in order to forecast CNV more consistently through simple and interpretable computational algorithms.

In light of the aforementioned factors, we here present a novel method for detecting CNV in NGS data, named OTSUCNV (based on OTSU). The idea is to use a straightforward and efficient sliding window strategy to locate breakpoints in RD data, and then use the OTSU method on the tiny data that has been processed to automatically isolate the anomalous portion. The two important contributions that we make are as follows:

1. A simple dynamic sliding window model is used to process the RD data so that base sequences in adjacent positions with similar RD values are merged, and breakpoints are identified.
2. The combination of the T-test and the adapted OTSU algorithm for the categorization of copy number abnormal and normal events allows for the correct identification of even low amplitude variant events with high confidence.

## Methods

### Overview of the OTSUCNV

**Fig. 1:**
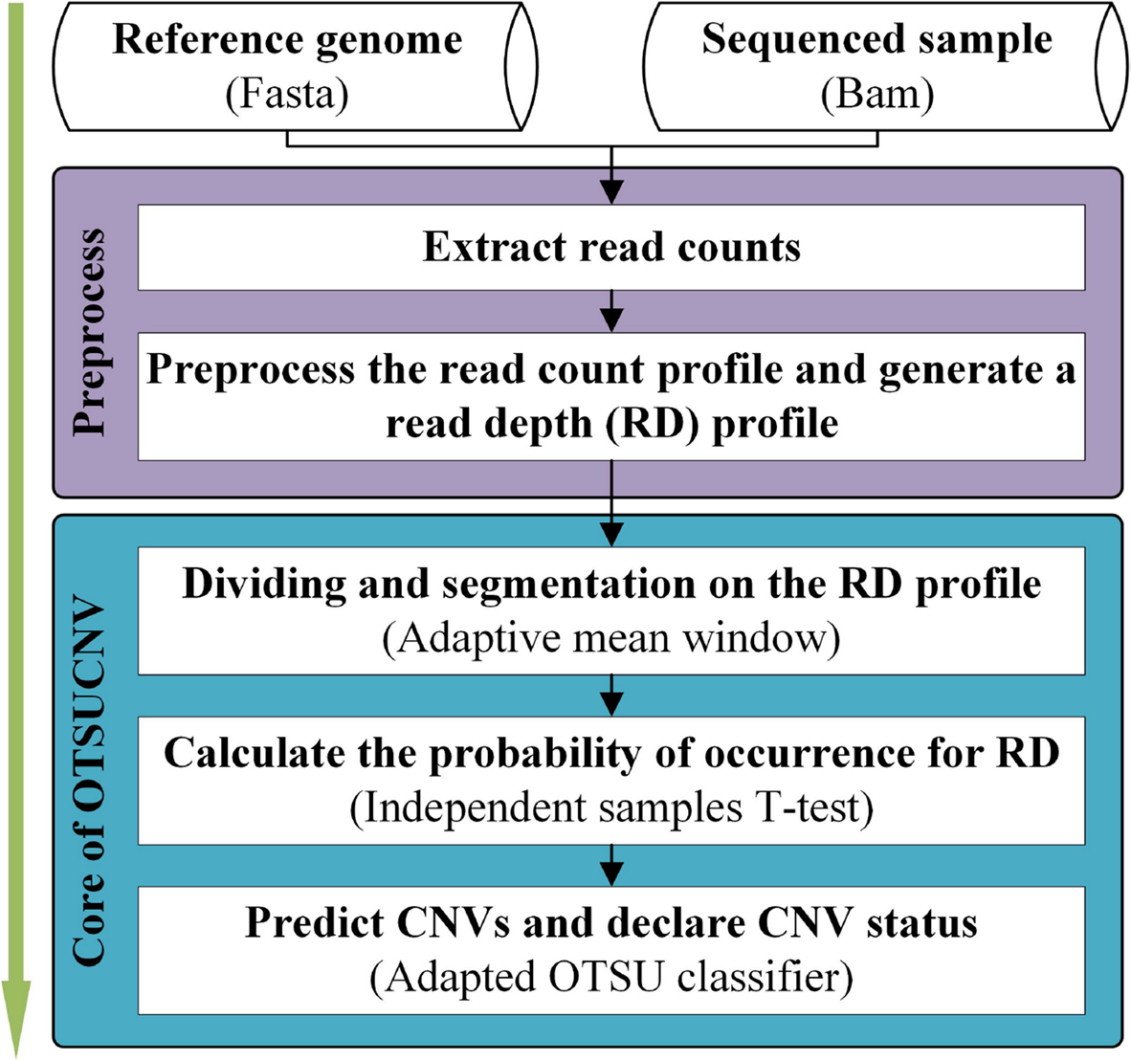
Flowchart of the OTSUCNV method

Figure 1 depicts the method’s workflow. It accepts a fasta-formatted reference sequence file and a bam-formatted read segment alignment file, preprocesses the input data, and then executes two primary phases to declare the CNV, including:

1. The entire DNA sequence is divided into contiguous and non-overlapping segments using the boxplot threshold and the adaptive mean window calculator proposed in this paper.
2. Using the independent samples T-test combined with the OTSU algorithm, CNV was inferred from the deviation between each segmental profile and the normal profile.

In addition, the method is implemented in Python and is available for free at https://github.com/hotsnow-sean/OTSUCNV.

### Preprocessing

Based on the input BAM alignment files, we can obtain the read count (RC) profile by tallying the number of read segments aligning to each position of the reference sequence, representing the coverage of each base position. Subsequently, we binning the reference genome into non-overlapping bin windows, and compute the average read count for each bin window, which is referred to as the read depth (RD) profile. Following the acquisition of the initial RD profile, some preprocessing will be applied to it, including eliminating reference genome unlawful bases and correcting GC bias, the latter of which uses a technique developed in earlier work [7, 12]. The RD profile needed for further processing will be obtained after the preprocessing. The RD profile can be written as follows, where *N* stands for the number of bins:

1 $$R=[r_1,r_2,\cdots ,r_N]^T\in \mathbb {R}^{N\times 1}$$

where $r_i$ represents the RD value of each bin.

### Segmentation

The pre-processed data are now ready to perform the segmentation procedure in order to identify contiguous regions with the same copy number (similar read depth values). In this paper, we propose an adaptive sliding window algorithm to accomplish the segmentation task, which determines the possible breakpoint locations based on the robustness mean difference between the local left and right sides, and then merges adjacent bins with similar RD values into larger segments. The algorithm is briefly described as follows.

First, for the sequence *R* to be processed, inspired by other researches [21, 22], we define the local one-sided robustness mean at a position *i* as

2 $$u_i^{right}=\frac{\sum \nolimits _{m=i}^k{\omega _{m,i}r_m}}{\sum \nolimits _{m=i}^k{\omega _{m,i}}}$$

where $\omega _{m,i}$ represents the weight of position *m* relative to the computational point *i*, theoretically, if the point *m* belongs to the same segment as the point *i*, then the weight $\omega _{m,i}$ is large, otherwise, it is small. In addition, *k* represents the maximum value of the one-sided size of the sliding window, which is used to limit the amount of calculation when many consecutive points belong to the same segment, and can be artificially specified. And *k* is much smaller than the size *N* of the RD profile.

To make the weight assignments reasonable, we use the following formula:

3 $$\omega _{m,n}=\mathrm{e}^{-\sum \nolimits _{i=n}^{m}{(r_i-r_{i-1})^2}}$$

A negative power function is used to achieve the purpose of decreasing the weights as the distance from the calculation point increases, where $r_i-r_{i-1}$ allows the weights to keep decreasing smoothly and slowly while the breakpoint is not crossed, and once the breakpoint is crossed, the significant difference in the RD values at the breakpoint will result in a significant decrease in the weight of subsequent calculations. According to this formula, due to the low weight of points from different segments, the resulting mean will better represent the average RD value of the same segment as the calculated points. In addition, if the weight $\omega$ is less than a certain threshold during the computation, the computation will be terminated directly to improve the computation efficiency and the robustness of the mean value. The threshold can also be specified artificially.

Along with the local mean, we define the local robustness mean difference as:

4 $$Diff_i=u_i^{right}-u_{i-1}^{left}$$

We calculate $Diff_i$ at each position in the sequence *Diff* according to the above equation to obtain a sequence of mean differences, denoted as *Diff*. In the sequence *Diff*, the values at the breakpoints will form extremes concerning the values on both sides of them. To mitigate the impact of small local extrema on the algorithm, here we use a boxplot procedure to filter out regions with relatively large values in *Diff*. The formulas to calculate the upper and lower bounds are as follows:

5 $$\begin{aligned} upperLimit &= Q3 + 3.5 \times IQR\nonumber \\ lowerLimit &= Q1 - 3.5 \times IQR \end{aligned}$$

*Q*1, *Q*3, and *IQR* are all statistical parameters of *Diff*. *Q*1 represents the first quartile, *Q*3 is the third quartile, and *IQR* is the difference between *Q*1 and *Q*3. By using the upper and lower bounds provided by the boxplot, we can efficiently filter out the relatively large and small values within a dataset.

For the filtered larger values as well as the smaller value regions, we further filter the local extreme or minimal values among them, and their locations are the breakpoints.

For the hyperparameters mentioned in the above algorithm, in addition to being artificially specified, a better parameter selection strategy has been derived in this study through extensive experiments, and the user can simply ignore the specification of parameters and use the default implementation in the provided program. The pseudo-code of the algorithm is shown below (Algorithm 1).

**Figure:**
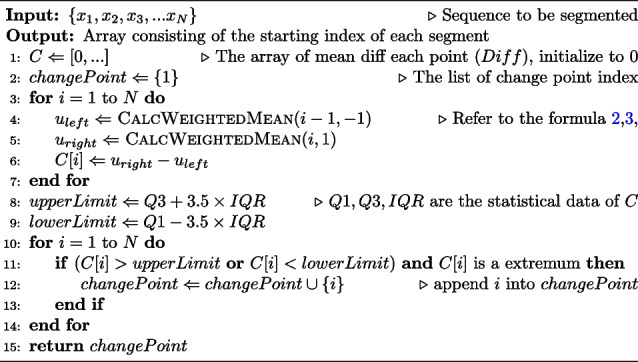
**Algorithm 1** Segment point detection

From Algorithm 1, it can be seen that the computational workload of this algorithm mainly focuses on the calculation of the robustness mean at each position. The calculation formula (2) for the robustness mean requires the computation of the weights of neighboring points. In the process of calculating the weights (formula 3), the sum of squared distances can be accumulated during the loop. Therefore, the complexity of calculating each neighboring point is *O*(1). As the number of points calculated around each point is significantly smaller than the scale of the RD profile, the overall computational complexity can be regarded as *O*(*N*). Therefore, the proposed algorithm can accomplish the segmentation task with a relatively low and stable time complexity.

After the segmentation, we partition *R* into some consecutive non-overlapping segments of different sizes according to the segmentation result, expressed by the following equation.

6 $$S=[s_1,s_2,\cdots ,s_n]$$

where $s_i$ represents the set of all RD values for the *i*-th segment.

**Fig. 2:**
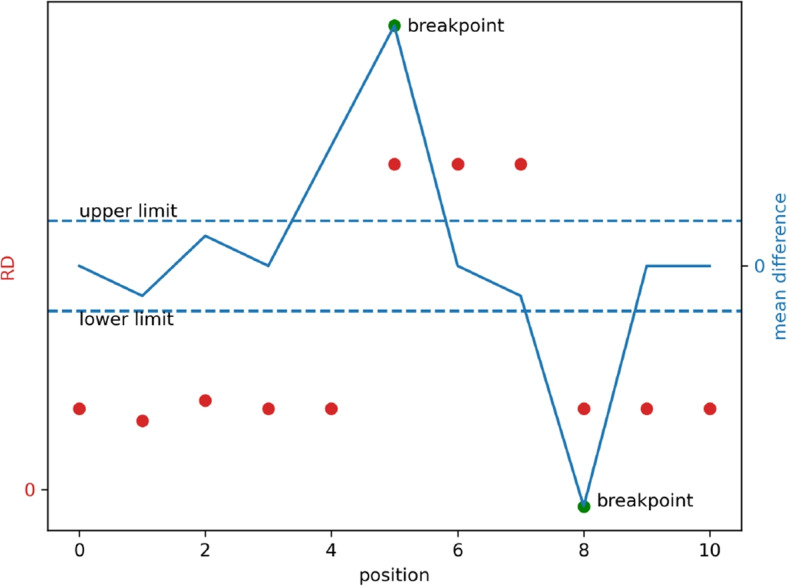
Example of Segmentation

To achieve a clearer understanding of the algorithm steps, we have provided a simple diagram in Fig. 2. The x-axis in the figure represents the position index, and the red point represents the RD values, while the blue line represents the calculated mean difference (formula 4). The two horizontal dashed lines represent the upper and lower limits, and the two green points represent the breakpoints obtained in the end. From the Fig. 2, it can be observed that the robust mean difference calculated using the proposed formula can effectively reflect the probability of a point being a breakpoint. After filtering with the threshold of the boxplot, reasonable inferences can be made regarding the potential location of breakpoints.

### Inferring CNVs based on the OTSU

#### Generating probability

Based on the set *S* obtained by the segmentation procedure, the RD values need to be initially classified according to their numerical magnitude. The probability of occurrence of each category of RD values must be evaluated before applying the OTSU algorithm. Therefore, the independent samples T-test is used here to test all the $s_i$ two-by-two pairs, and the sets that are not significantly different are aggregated into the same class. This results in several categories of RD values, and assuming a total of *m* categories, each category can be expressed as the set of several segments that are not significantly different from each other:

7 $$\begin{aligned}&C_i = [s_*,...] \nonumber \\&\sum \limits _{i=1}^m{|C_i|}=\sum \limits _{i=1}^m{n_i}=n \end{aligned}$$

where $|C_i|$ and $n_i$ denote the number of elements in $C_i$ and *n* denotes the number of elements in the set *S*.

We take the average value of the RDs contained in each class as a representative value, and the ratio of the number of its elements to the number of all segments as the probability of the occurrence of this RD value. Then for a certain average value of RD, the probability corresponding to it is as follows:

8 $$\begin{aligned} P(u_i)=\frac{|C_i|}{n}=\frac{n_i}{n}, u_i=\frac{\sum \nolimits _{s_*\in C_i}RD_{s_*}}{n_i} \end{aligned}$$

#### Predicting CNVs by OTSU

After the previous processing, the average RD value of each category and its corresponding probability of occurrence can be obtained. Next, to distinguish the abnormal segments from normal segments, we use the OTSU [23] method, which is an application method for the automatic selection of thresholds in the field of image segmentation with simple computation and good self-adaptability and can find a threshold with high confidence according to the distribution of the data itself.

First, for copy number variation detection, we can consider the abnormal event as the foreground and the normal event as the image’s background. Since copy number abnormalities can be simply divided into two types of numerical performance: increasing and missing, in order to unify the processing, we find the distance of all RD values to the RD values corresponding to the normal copy number and obtain the following distance array (the distances are listed in ascending order):

9 $$\begin{aligned} D&=abs([u_1,u_2,\cdots ,u_m]-u_{normal})\nonumber \\&=[x_1,x_2,\cdots ,x_m], x_1\le x_2\le \cdots \le x_m \end{aligned}$$

where $u_{normal}$ denotes the RD value corresponding to the normal copy number, which can be calculated by any reasonable method, the most common method is to take the plural. In this paper, we use a more robust method to calculate it, this method can be referred to [24]. For convenience, we denote the previously obtained probabilities as:

10 $$\begin{aligned} &P=[p_{1},p_{2},\cdots,p_{m}]\\ & \sum\limits_{i=1}^{m}{p_i}=1\end{aligned}$$

Note that the index of the probabilities indicated above corresponds to the index of the distance values.

According to the characteristics of the RD-based method, the larger the deviation from the normal RD value, the more abnormal the segments corresponding to that RD value are. Now suppose that the data are divided into two categories $C_0$ and $C_1$ by a certain threshold *k* where $C_0$ represents the category less than or equal to the threshold and $C_1$ represents the category greater than the threshold, then the probability of occurrence of each category and the respective mean values are given by the following equation:

11 $$\begin{aligned}&\omega _0=\sum \limits _{i=1}^k{p_i}=\omega (k) \\&\omega _1=\sum \limits _{i=k+1}^m{p_i}=1-\omega (k) \\&\mu _0=\sum \limits _{i=1}^k{x_i}\Pr (x_i|C_0)=\sum \limits _{i=1}^k{x_ip_i/\omega _0}=\mu (k)/\omega (k) \\&\mu _1=\sum \limits _{i=k+1}^m{x_i}\Pr (x_i|C_1)=\sum \limits _{i=k+1}^m{x_ip_i/\omega _1}=\frac{\mu _T-\mu (k)}{1-\omega (k)} \end{aligned}$$

where

12 $$\begin{aligned}&\omega (k)=\sum \limits _{i=1}^k{p_i}\\&\mu (k)=\sum \limits _{i=1}^k{x_ip_i}\\&\mu _T=\mu (m)=\sum \limits _{i=1}^m{x_ip_i} \end{aligned}$$

The between-class variance is defined as:

13 $$\begin{aligned} \sigma ^2&=\omega _0\cdot (\mu _0-\mu _T)^2+\omega _1\cdot (\mu _1-\mu _T)^{2}\\&=\omega _0\cdot \omega _1\cdot (\mu _1-\mu _0)^{2}\\&=\frac{(\mu _T\cdot \omega (k)-\mu (k))^2}{\omega (k)\cdot (1-\omega (k))} \end{aligned}$$

After that, by searching the optimal threshold *k* that maximizes the between-class variance, the normal and abnormal data can be separated.

**Figure:**
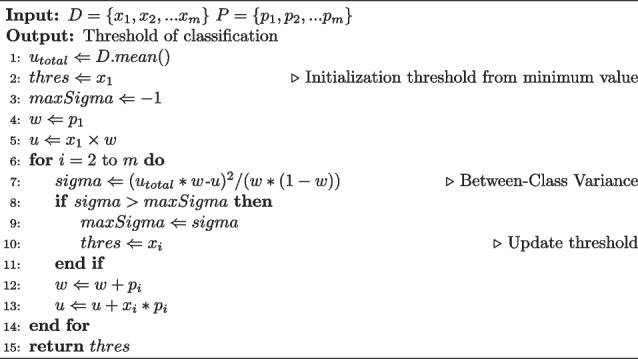
**Algorithm 2** OTSU classifier

According to Algorithm 2, it can be seen that the OTSU classifier only has a single loop of *m* iterations, therefore the time complexity of this algorithm is *O*(*N*), which is linear complexity.

In addition, owing to the peculiarity of CNVs, the magnitude of the gain fragment can be much larger compared to the loss fragment. This can cause an imbalance in the distribution, which can be similar to uneven lighting in image segmentation which can have a significant impact on the performance of the OTSU algorithm. Here we use a simple strategy to reduce the negative impact of this situation on the prediction results, called extreme value suppression. In brief, before applying the OTSU algorithm, we reduce the values in the distance array *D* that are too large, using the following formula:

14 $$\begin{aligned} D(i) = \left\{ \begin{array}{ll} D(i), &{} D(i) <= D_{mean} \\ D_{mean}, &{} D(i) > D_{mean} \end{array}\right. \end{aligned}$$

where $D_{mean}$ represents the average of all distance values. In theory, if the above-mentioned extreme values exist, then this step will affect just the data closest to the extreme values, achieving the goal of limiting the negative effects of the extreme values. If there is no such extreme value, the distance values corresponding to all anomalous RDs are equally reduced and have little effect on the prediction outcomes. In the subsequent sections, we will illustrate the effectiveness of this procedure with experimental results.

**Fig. 3:**
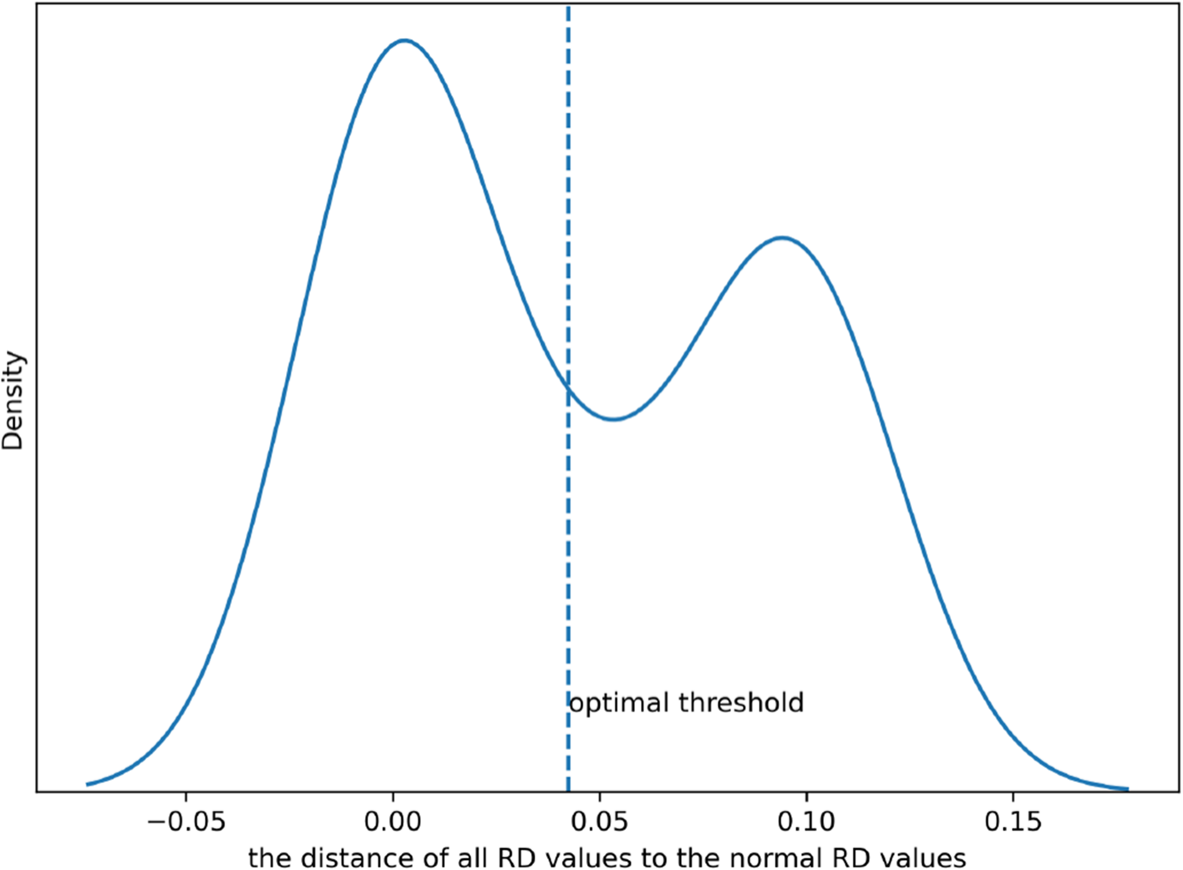
Example of finding the optimal threshold using OTSU

In order to provide a clearer understanding of the algorithm steps, we have presented an example in Fig. 3. The x-axis in the figure represents the distance of all RD values relative to the normal RD value (refer to formula 9), while the y-axis represents the probability density of the values. And the dashed vertical line indicates the position of the optimal threshold calculated by the OTSU algorithm. From the probability density curve of the data distribution in Fig. 3, it can be observed that all the data are mainly concentrated in two peaks, with the peak near the position close to 0 (corresponding to normal RD values) being higher. The optimal threshold calculated by the OTSU algorithm is precisely located near the valley where the two peaks intersect. This clearly demonstrates the reliability of differentiating between normal and abnormal data using the OTSU algorithm.

## Results

To assess the effectiveness of OTSUCNV, we performed experiments on both simulated and real datasets. For each dataset type, we compared our proposed method with four peer methods designed for the same purpose. Furthermore, the efficacy of these methods was measured using precision, sensitivity, and F1-score metrics. Precision was defined as *TP*/*PP*, sensitivity as *TP*/*P*, and F1-score as the harmonic mean of precision and sensitivity. In this context, *TP* refers to the number of genomic positions that are duplicated both in the declared CNVs and the confirmed CNVs. *PP* corresponds to the total number of genomic positions included in the declared CNVs, whereas *P* represents the total count of positions in the confirmed CNVs.

### Simulation studies

For the simulated experiments, we utilized hg38 as our reference genome, which is available for download from the Ensembl database, http://asia.ensembl.org/. Then, we simulated the test gene sequences using SInC [25] and ART [26], along with a reference genome. In this study, SInC was responsible for simulating copy number variations in the normal reference sequence, while ART was used to simulate sequencing of the generated test sequences and ultimately produce FastQ files [27]. Subsequently, BWA [28, 29] and Samtools [30] were used with default parameters to obtain the aligned BAM file for CNV detection. In this study, we used SInC to generate three different sets of gene sequences with CNV region lengths ranging from 3000 to 50000 bp. For each sequence, ART was employed to generate sequencing data with coverage depths of 2X, 6X, and 10X. To ensure the reliability of our experiments, each coverage depth was repeated 30 times to minimize experimental variability. Finally, the average performance of 90 samples was taken as the final metric for each of the three different sequencing coverages.

**Fig. 4:**
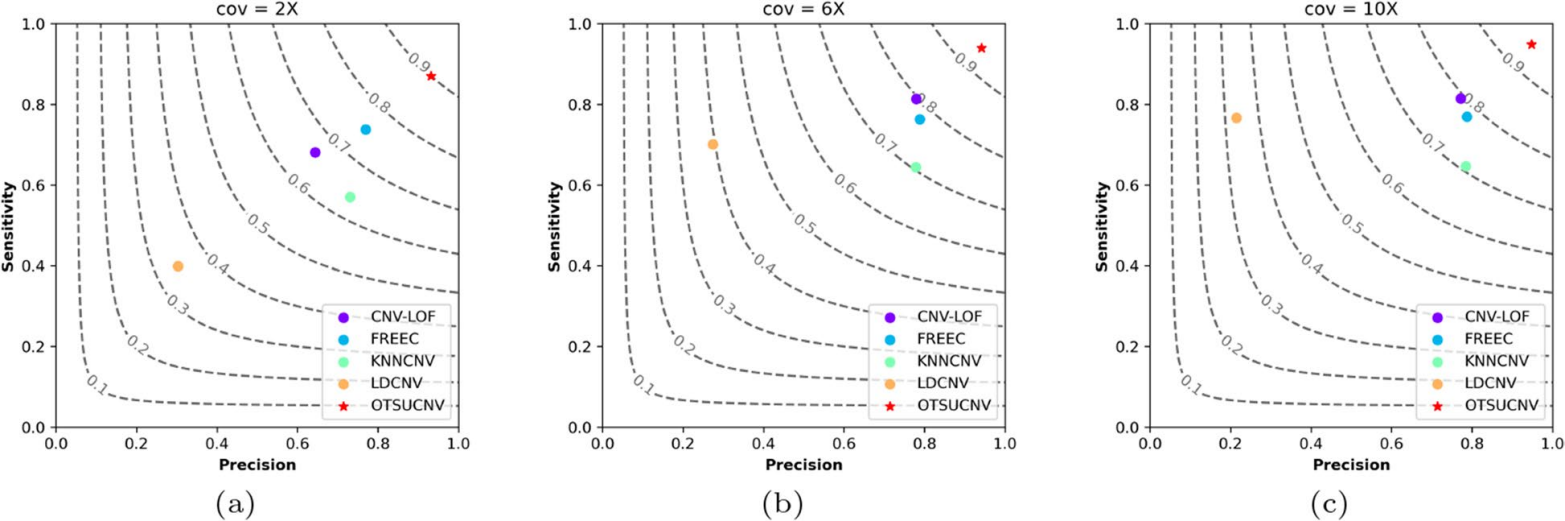
Performance comparison of OTSUCNV with the four peer methods in terms of precision, sensitivity, and F1-score. The F1-score is shown in black dashed lines ranging from 0.1 to 0.9 with an increment of 0.1. **a**-**c** They represent the performance of the aforementioned approaches for three distinct coverage samples: 2x, 6x, and 10x

Using the simulation data generated above, we compared its performance with four different peer methods, which are FREEC [11], CNV-LOF [15], KNNCNV [31], and LDCNV [20]. Figure 4 shows the experimental results of these methods on simulation data, where the experimental results for each different coverage are averaged over a total of 90 samples for 3 different variant configurations and 30 sequencing repetitions of the simulation. According to the figure, FREEC shows an F1 score close to 0.8 in samples with different coverage. LDCNV performs poorly in terms of precision, ranking fifth in F1 score. The F1 scores of CNV-LOF and KNNCNV improve with increasing sample coverage, ranging between 0.6 and 0.8. While our method outperforms the other four peer methods in terms of precision, sensitivity, and F1 score. Even in samples with 2x coverage, the F1 score remains around 0.9. Overall, OTSUCNV performs better than the other four peer methods on the simulated dataset.

**Fig. 5:**
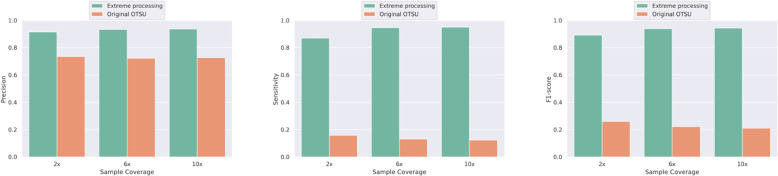
Comparison of the performance of the original OTSU and the OTSU after applying the extreme value suppression in terms of precision, sensitivity, and F1-score

To further discuss the importance of the extreme value suppression in the proposed method, we conducted an ablation experiment with the same experimental data and experimental steps for the extreme value suppression step. The experimental results are shown in Fig. 5, and it can be seen that the application of this step led to a significant increase in the sensitivity of the CNV prediction, thus greatly improving the F1 score of the results.

### Application to real datasets

The real sequencing samples were obtained from the 1000 Genomes Project [32]. For our study, we selected six commonly used samples (NA12878, NA12891, NA12892, NA19238, NA19239, NA19240) in this field of research, all of which were aligned to the hg18 version of the reference sequence. In this algorithm study, these six samples were only used for tool performance validation. The DGV Gold Standard Variants for these samples were downloaded from the Database of Genomic Variants (DGV, http://dgv.tcag.ca/dgv/app/home) [33]

**Fig. 6:**
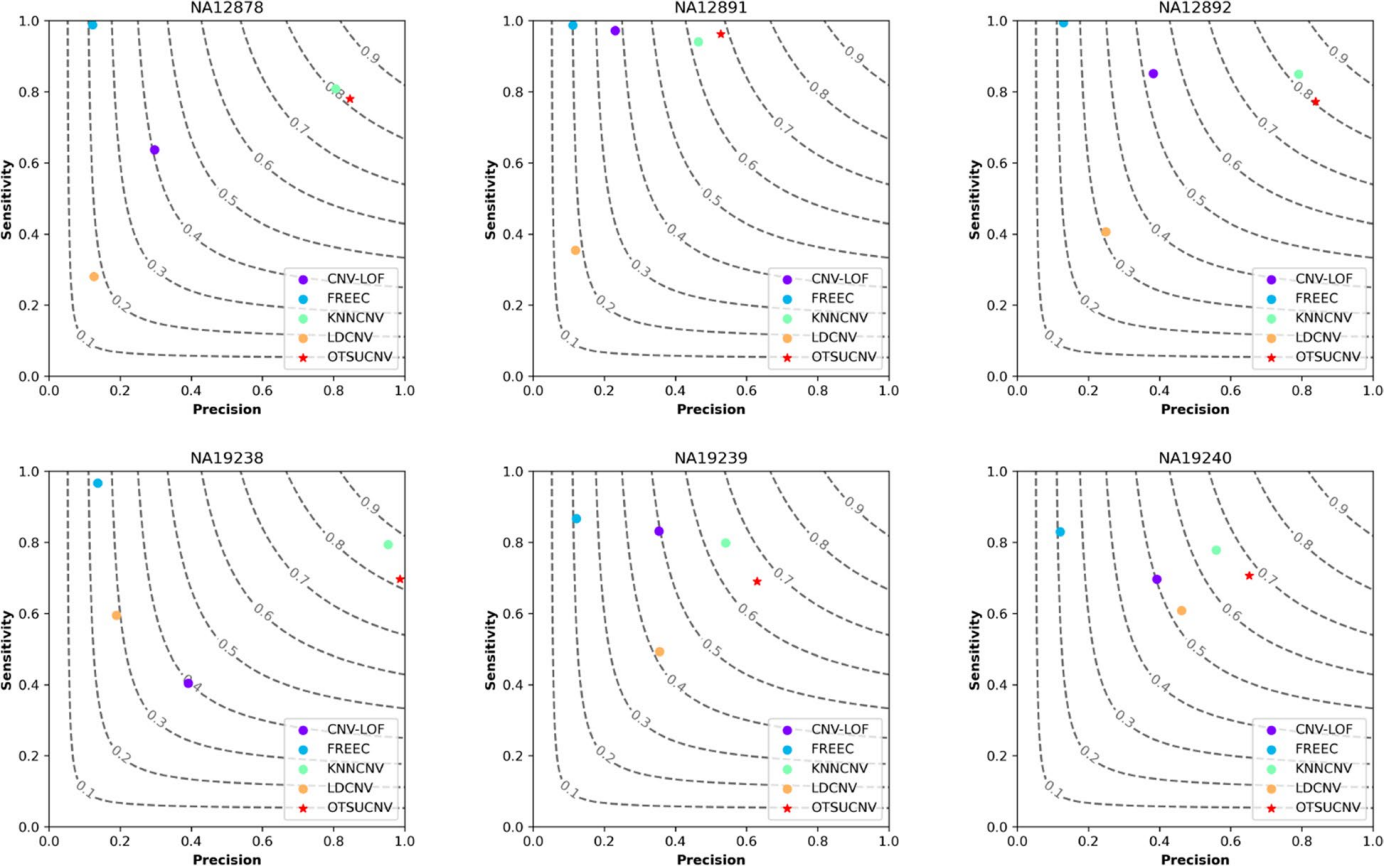
Performance comparison of OTSUCNV with the four peer methods in terms of precision, sensitivity, and F1-score. The F1-score is shown in black dashed lines ranging from 0.1 to 0.9 with an increment of 0.1. The six subplots show the performance of these methods on each of the six real data sets

As shown in Fig. 6, we conducted comparison experiments with four previous peer methods on six real datasets. Based on the experimental results, our proposed method achieves a relatively high level of F1 score. Specifically, CNV-LOF, FREEC, and LDCNV have lower overall rankings due to their lower precision. In comparison to KNNCNV, OTSUCNV demonstrates higher F1 scores on four samples and exhibits higher precision on each sample. Overall, OTSUCNV also demonstrates advantages in experiments with real samples.

### Comparison of running time

To evaluate the execution efficiency of the algorithm, the proposed method was tested on 30 simulated samples along with four peer methods. The tests were conducted on a PC with a 2.9GHz CPU and 16.0GB memory. The average execution time for the 30 samples is shown in the Table 1.

**Table 1:** Comparison of running time of five methods

Method	CNV-LOF	FREEC	KNNCNV	LDCNV	OTSUCNV
Running time (s)	19.4396	6.4904	12.9317	17.1584	12.7013

In terms of execution time, our method is the fastest, except for FREEC. However, FREEC requires additional preprocessing to calculate the percentage of GC content in a given sequence file in FastA format, and its test time does not include the time for GC calculation. The step took approximately 8 seconds under the same experimental conditions. Overall, OTSUCNV is an efficient CNV detection approach.

## Discussion and conclusion

We developed a novel method for CNV detection in whole genome sequencing, called OTSUCNV, which has been demonstrated to perform well on samples of different coverage depths and both real and simulated datasets. We can apply it to the analysis of germline and tumor data. OTSUCNV first segments DNA sequences using an adaptive sliding window technique, and then clusters the segmented RDs using independent sample T-tests to obtain the probability of occurrence of each class of RDs. Finally based on the OTSU algorithm, all RD representative values are classified as normal or abnormal, and the gene segments they correspond to are naturally indicated as CNVs. Our method has several advantages: (1) the proposed sequence segmentation approach exhibits good breakpoint detection performance in RD-based methods; (2) the use of the modified OTSU method for CNV prediction eliminates the difficulty of manually selecting thresholds and demonstrates good performance both theoretically and practically; (3) compared to four peer methods, our algorithm has low computational time complexity, with segmenting and predicting stages having only linear time complexity. Overall, our method offers high cost-effectiveness in CNV detection.

We conducted studies with four peer approaches on both simulated and real datasets to illustrate the effectiveness of the OTSUCNV method. The experimental results show that our method outperforms other four methods in terms of F1 scores, outperforming them comprehensively on simulation datasets and performing similarly to KNNCNV on real datasets. Moreover, through a comparison of running times, is has been proven that OTSUCNV is more efficient. Therefore, OTSUCNV may become a promising tool for detecting CNVs.

For future work, we plan to make improvements to our method in the following two areas: (1) In the RD-based CNV detection method, the size of the RD calculation window is a crucial factor but currently, the selection is based on empirical knowledge. Therefore, we intend to design an algorithm to avoid manual selection. (2) During the CNV prediction stage, we treat both gain and loss cases as the same anomalous event. Although the impact of this strategy is currently reduced by the method of extreme value suppression, it should be possible to find a more robust treatment. Therefore, we plan to further optimize the algorithm to solve this problem.

## Data Availability

The data used in the research are explained in the paper, further inquiries can be directed to the corresponding author.
